# Case Report: Ketogenic Diet Is Associated With Improvements in Chronic Obstructive Pulmonary Disease

**DOI:** 10.3389/fmed.2021.699427

**Published:** 2021-07-29

**Authors:** Nicholas G. Norwitz, Russell Winwood, Brianna J. Stubbs, Dominic P. D'Agostino, Peter J. Barnes

**Affiliations:** ^1^Department of Nutrition, Harvard Medical School, Boston, MA, United States; ^2^Respiratory Network, Ministry of Health Agency for Clinical Innovation, St Leonards, NSW, Australia; ^3^Buck Institute for Research on Aging, Novato, CA, United States; ^4^Morsani College of Medicine, University of South Florida, Tampa, FL, United States; ^5^Institute for Human and Machine Cognition, Pensacola, FL, United States; ^6^National Heart and Lung Institute, Imperial College, London, United Kingdom

**Keywords:** chronic objective pulmonary disease, ketogenic diet, forced expiratory volume 1, inflammation, case report

## Abstract

Chronic Obstructive Pulmonary Disease (COPD) is a debilitating inflammatory respiratory condition that presents with worsening breathing difficulties and it is assumed to be progressive and incurable. As an inflammatory disease, COPD is associated with recruitment of immune cells to lung tissue and increased levels of pro-inflammatory cytokines, including TNF-α, IL-1β, IL-6, IL-8, and GM-CSF. Low-carbohydrate ketogenic diets have anti-inflammatory properties that could, in theory, improve COPD symptoms and progression. Herein, we report on a 54-year-old patient (C.A.) with COPD who adopted a ketogenic diet (70% calories from fat). Subsequently, C.A. experienced a reduction in inflammatory markers in association with a meaningful improvement in lung function. His inflammatory markers decreased into the normal range and his forced expiratory volume increased by 37.5% relative to its pre-ketogenic diet value. Future research should explore nutritional ketosis and ketogenic diets as possible therapeutic options for individuals with COPD.

## Introduction

Chronic Obstructive Pulmonary Disease (COPD) affects over 300 million patients worldwide and is currently the third ranked cause of death globally (1). COPD is characterized by slowly progressive airflow limitation as a result of peripheral airway obstruction (chronic bronchiolitis) and lung parenchymal destruction (emphysema), which lead to increasing shortness of breath on exertion (2). COPD is further associated with inflammation of the lung, including the recruitment of macrophages, neutrophils and lymphocytes and the secretion of multiple inflammatory mediators, including cytokines such as TNF-α, IL-1β, IL-6, IL-8, and GM-CSF (3). This pulmonary inflammation is not reduced by the mainstay of current therapy, inhaled long-acting bronchodilators, and is also largely resistant to corticosteroids (4). Targeting of individual cytokines has been unsuccessful, reflecting the fact that many mediators are involved in pathogenesis of the disease (5). Importantly, there are no current therapies that significantly improve disease progression. Therefore, there is a pressing need to find broader spectrum anti-inflammatory treatments for COPD that will improve symptoms, disease progression, and patient quality of life (6).

Ketogenic diets (KD) have a near century long history of being used to treat pediatric epilepsy (7), and newer research is beginning to explore their therapeutic potential in other chronic disease, such as type II diabetes (8, 9), polycystic ovarian syndrome (10), Alzheimer's disease (11), Parkinson's disease (12), cardiovascular risk (13), metabolic syndrome (14), and various mental illnesses (15). Many of these disease have an inflammatory component and, correspondingly, well-formulated ketogenic diets have been shown to improve a broad spectrum of inflammatory markers (16).

The anti-inflammatory effects of KD may be mediated, in part, by the ability of the ketone molecule, β-hydroxybutyrate (βHB), to inhibit the NLRP3 inflammasome (17, 18). NLRP3 is a protein complex that positively regulates the inflammatory response, and inhibition of NLRP3 is a mechanism whereby βHB is thought to mitigate inflammatory conditions such as gout (19). NLRP3 is also elevated in active COPD, as measured by circulating and local levels of NLRP3, Asc, and caspase-1 mRNAs (20). These mechanistic data, along with the clinical data mentioned above, suggest that a KD could have a beneficial effect in COPD patients, possibly by inhibiting NLRP3.

With respect to pulmonary diseases, KD improve symptoms in asthma (21) and trials are ongoing to determine whether KD may protect against severe COVID-19 disease, including lung disease (22). Importantly, a 3-week controlled trial including 60 COPD patients demonstrated a small but significant improvement in forced expiratory volume in 1 s (FEV_1_) with a lower carbohydrate group (47% calories from carbohydrates) compared to a higher carbohydrate group (65% calories from carbohydrates) (23). In this study 10% of calories in the lower carbohydrate group were obtained from medium chain triglycerides to induce mild ketosis.

While COPD is an inflammatory disease and KD are known to be anti-inflammatory, there are no reports of KD being used to treat COPD existing in the medical literature. Herein we report on such a case in which an individual with COPD adopted a KD and subsequently observed improvements in inflammatory markers and lung function.

## Case Description

The subject of this study (C.A.) is a 54-year-old male in whom COPD was diagnosed in 2011, at age 45 years. Early in childhood, at age 10, C.A. was diagnosed with asthma. He also smoked cigarettes from age 17 and to 37 (~15 pack-years) and his grandfather died of lung cancer at age 67. C.A.'s only co-morbidity was and remains asthma, and he is free of other common comorbidities of COPD, including diabetes, pre-diabetes, cardiovascular disease, osteoporosis, sleep or mood disorders, metabolic syndrome, or obesity.

In year prior to diagnosis, C.A.'s chief complaints were worsening shortness of breath and more frequent chest infections. In 2011, spirometry showed an FEV_1_ of 0.79L (22% predicted normal) with an FEV_1_/FVC ratio of 40%. There was an increase in lung volume measured by body plethysmography, with an increase in air trapping, but there was no reduction in gas transfer. There was no significant bronchodilator response to inhaled salbutamol, nor any response to a trial of oral corticosteroids (prednisolone 40 mg od for 4 weeks). A computerized tomography scan showed no evidence of emphysema, indicating that the COPD is due to small airway disease. C.A. was diagnosed with very severe COPD (GOLD stage 4) and was treated with inhaled tiotropium bromide once daily and budesonide-formoterol combination twice daily, which he has continued. He has also taken zinc and vitamin C supplements daily since his diagnosis in 2011.

Over the ensuring 4 years C.A. undertook an aerobic exercise program consisting of daily running, cycling or swimming and ate a “balanced diet” consisting of fats, proteins, and carbohydrates, including fruits, vegetables and whole grains. In association with his daily activity and diet, his BMI decreased (from 29.4 kg/m^2^ at time of diagnosis in 2011 to 23.8 kg/m^2^ in 2017), his exercise maximal peak capacity increased from 165 to a peak of 193 watts, as measured by cycle ergometer, and his FEV_1_ improved only marginally to 0.91L (25% predicted normal).

In 2017, he began a low-carbohydrate, high-fat diet KD. The macronutrient composition of the diet comprised calories from 70% fat, 20% protein, and 10% carbohydrates and C.A. confirmed nutritional ketosis of >0.5 mmol/L daily by fingerstick using an Abbott Optium Neo device that measured blood D-βHB. While on the KD, he lost no more weight, remaining at a BMI of 23.8 kg/m^2^ at the time of the 2019 and 2020 measurements listed below.

**Figure 1:**
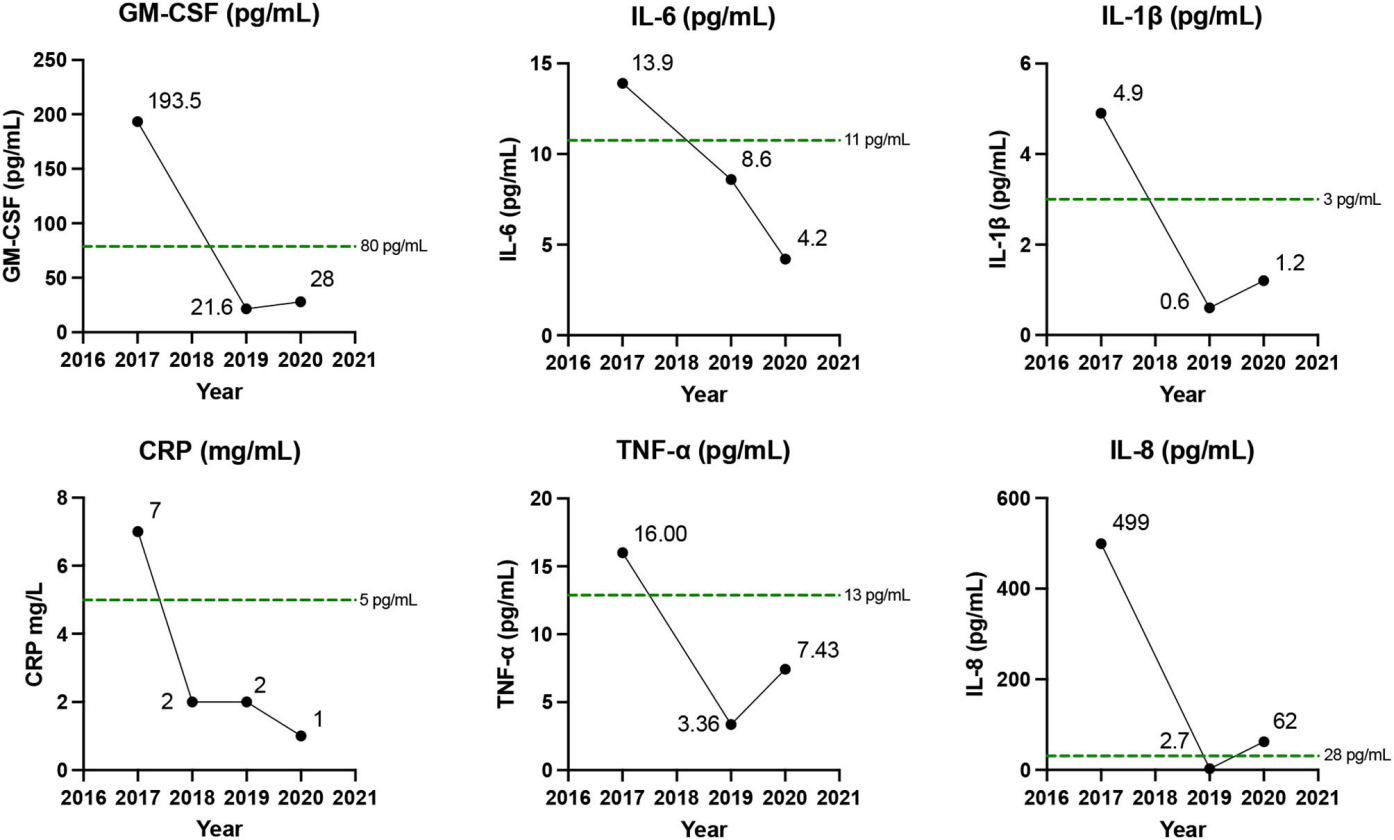
Inflammatory biomarkers. Data represent C.A.'s inflammatory biomarkers prior to starting a ketogenic diet and at two time points, 2019 and 2020, after commencing the diet. Green dotted line represent the threshold of normal range for each biomarker. All markers improved on a ketogenic diet.

Prior to starting the KD, baseline plasma TNF-α, IL-1β, IL-6, IL-8, and GM-CSF, concentrations were 16, 4.9, 13.9, 499, and 193.5 pg/mL, respectively, and CRP was 7 mg/L. All measurements were above the upper threshold of normal. In 2019, 2 years into his KD, blood concentrations decreased to 3.4 (TNF-α), 0.6 (IL-1β), 4.2 (IL-6), 2.7 (IL-8), and 21.6 pg/mL (GM-CSF), and CRP was 1 – 2 mg/L (Figure 1). These measurements were repeated in 2020, with maintenance of the KD, and results were similar. On both occasions all post-KD inflammatory markers were within the normal range with the singular exception of the 2020 IL-8 (62 pg/mL), although this measure still represented an 8-fold decrease from its pre-KD level. All three cytokine panels were ordered direct to consumer (NutriPath) by C.A. The 2017 baseline test was ordered for interest following his self-study on COPD. At this time, there was no thought of a case report; however, C.A.'s response to dietary change was—in his perspective (below)—so remarkable that, in 2019, he decided he wanted to collect follow-up data to correlate with his improved symptoms and quality of life out of interest. He also ordered the 2020 tests as a replicate.

**Figure 2:**
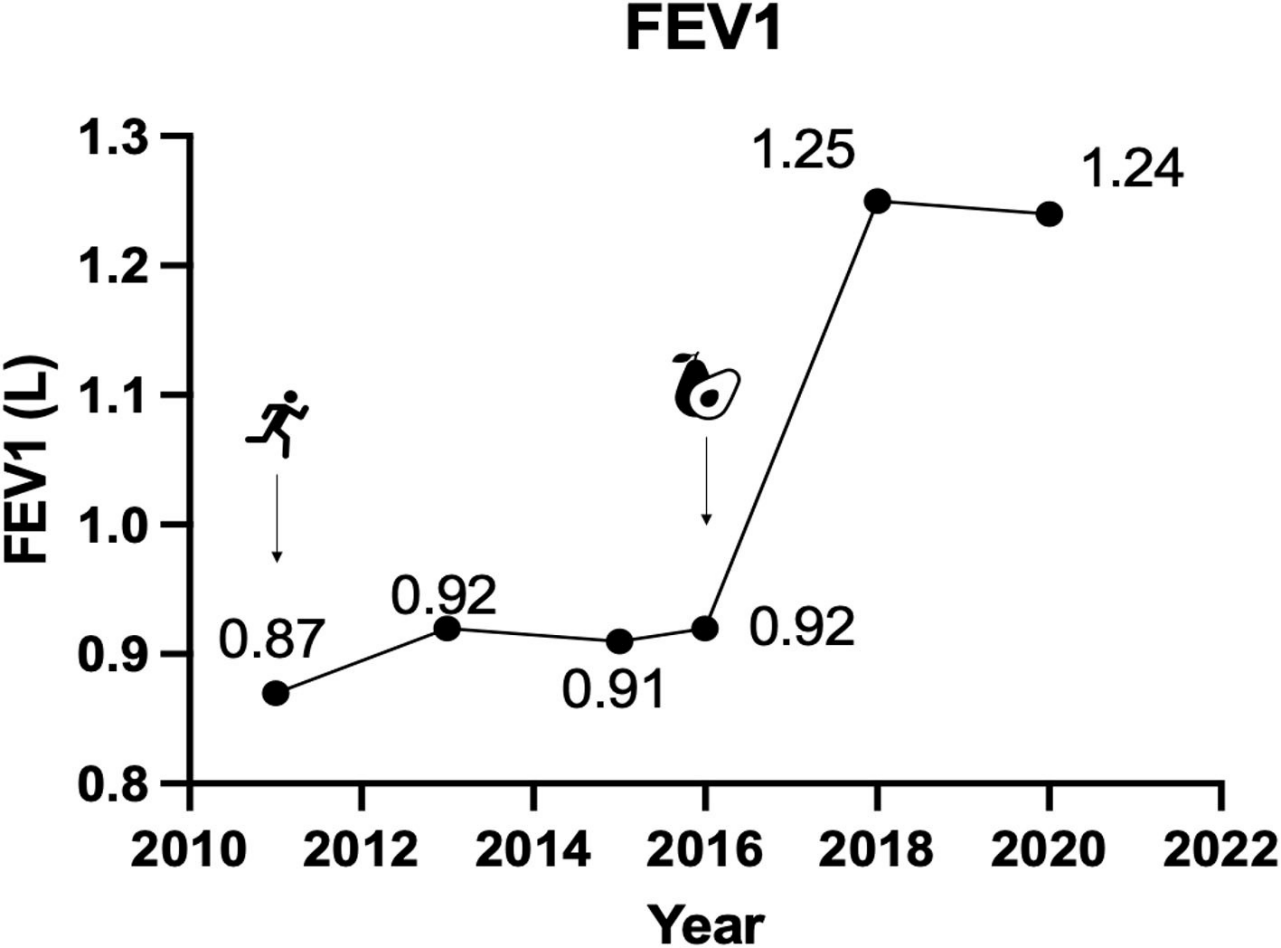
Forced expiratory volume in 1 s. Data represent C.A.'s FEV1 at four time points prior to starting a ketogenic diet and at two time points, 2019 and 2020, after commencing the diet.

In conjunction with the improvement in inflammatory blood biomarkers (Figure 1), C.A.'s FEV_1_ improved to 1.24 (35% predicted) and 1.25L in 2019 and in 2020, respectively, representing an improvement in FEV_1_ of 38% relative to pre-KD measurements (Figure 2). All lung function tests, including the baseline test, were administered 24 h after stopping bronchodilators. Prior to the diet he suffered from 1 to 2 acute exacerbations/year but reported no exacerbations since taking the KD. He also reported improvement in symptoms and quality of life, with reduced use of rescue salbutamol inhaler from 3 to 4 puffs daily before the KD to only one or less puffs on the diet. His exercise tolerance also improved markedly to the extent that he was able to complete marathons.

## Discussion

C.A. suffers from severe COPD, predominantly due to small airway disease and showed only modest improvement in symptoms and lung function with maximal inhaler therapy (inhaled corticosteroid, long-acting muscarinic antagonist and long-acting β_2_-agonist), as recommended by current management strategies (24). After switching to a KD, he had reduced symptoms and improved exercise tolerance, used less rescue inhaler and had no further acute exacerbations. His FEV_1_ improved by over 35%, relative to baseline, whereas there had previously been no significant improvement with a bronchodilator or a systemic corticosteroid. Furthermore, between 2011 and 2017, his self-prescribed exercise program increased his FEV_1_ from 0.79L (22% predicted normal) to FEV_1_ from 0.91L (25% predicted normal), in conjunction with a BMI decrease from 29.4 to 23.8 kg/m^2^. By comparison, the addition of a KD was associated with a much larger improvement in FEV_1_ to 1.24 and 1.25, in 2019 and 2020, respectively (~35% predicted), without any change in weight. As the KD improved C.A.'s exercise tolerance, it's possible that there was a synergy between the KD and exercise, i.e., that the KD acted as a therapeutic adjunct. It is also possible, and perhaps more likely, that the KD had an independent effect.

The clinical improvements C.A. experienced after adopting the KD were accompanied by a marked fall in plasma concentrations of several inflammatory cytokines known to be increased in COPD (TNF-α, IL-1β, IL6, IL-8, GM-CSF, and CRP) that were elevated prior to the diet. While the data on hand do not permit us to draw a causal conclusion that the KD improved C.A.'s COPD, the coincident drop in serum cytokines and improvements in FEV_1_, along with prior clinical and non-clinical literature demonstrating anti-inflammatory and therapeutic beneficial effects of a KD in inflammatory disorders, suggest that a KD could have potential for the treatment of COPD. This is an area that deserves more structured investigation.

Several dietary interventions have been evaluated in COPD, mainly with a view to increasing skeletal muscle mass as well as addition of nutraceuticals, such as antioxidant vitamins and vitamin D, but so far there is no convincing evidence for the efficacy of these diets, and they are not recommended in the routine management of COPD patients (25). Since diagnosis in 2011, C.A. only ever reported consuming a zinc and vitamin C supplement and their dosing did not change upon starting a KD. Dietary interventions may theoretically benefit the lung disease, but also the comorbidities that are commonly seen in COPD patients (26), including diabetes, pre-diabetes, obesity, ad metabolic syndrome, although C.A. presented with none of these conditions nor did the KD cause C.A. to lose weight. Furthermore, dietary interventions may be more acceptable to patients than long-term drug therapies, which have poor adherence in COPD (27). Conversely, despite the common conception that KD are not sustainable for patients, trials show that patients given adequate education and support adhere to and enjoy KD as much as other diets and standard of care (8, 28).

Although a KD has been shown to improve asthma, there are no previous reports of its use in COPD patients. Given the parallel decrease in cytokines and improvement in FEV_1_, it is possible, if not likely, that the direct anti-inflammatory effect of the KD mediate part of the therapeutic benefit of the lifestyle in this case. βHB is known to inhibit the NLRP3 inflammasome (17, 18), which plays a pathological role in COPD (20). Ketogenic diets have also been shown to lower a wide range of inflammatory cytokines and have been shown to outperform isocaloric low-fat diets in their ability to lower TNF-α, IL-6, and IL-8 (16) and reverse insulin resistance and metabolic syndrome (14), which are common inflammatory comorbidities associated with COPD (29, 30).

Another possible mechanism for the clinical improvement in COPD may be a change in the gut or lung microbiome. Lung microbiome is abnormal in COPD, and patients with severe COPD commonly have colonization of the lower respiratory tract with bacteria such as *Haemphilus influenzae, Streptococcus pneumoniae*, and *Moraxella cattarhalis* (31, 32). These colonizing bacteria may be important in inducing a chronic inflammatory response, with increases in cytokines TNF-α, IL-1β, IL-6, and IL-8. Correspondingly, mouse data suggest that changes in the microbiome mediate some of the therapeutic effects of ketogenic diet against epilepsy (33) and that KDs can alter the microbiome to protect against activation of Th17 cells and other inflammatory mediators (34).

An obvious limitation of this case study is that the data do not allow us to draw conclusions about the mechanism of action by which a KD improved inflammation and respiratory function in the patient, C.A. We can only note the striking association among the onset and maintenance of KD and concomitant improvements in all measured inflammatory markers and FEV_1_. Nevertheless, C.A.'s improvements make mechanistic sense in the context of the broader literature on COPD as an inflammatory disorder and KD as an anti-inflammatory intervention. Future animal model research should focus on exploring the mechanisms of action of KD on COPD and related respiratory conditions with a mind toward providing patients with a lifestyle option to treat disease and improve quality of life.

## Patient Perspective

After my COPD diagnosis, I made a commitment to myself that I would do anything I could to improve my health so took up running, swimming, and cycling. Even though, I wasn't very good, I especially loved distance running. I started listening to podcasts and reading about how nutrition could improve my breathing and endurance running performance. The information I gathered suggested a low-carb ketogenic diet could help burn fat as fuel more efficiently, I thought I'd give it a go. I was surprised to find my breathlessness diminished as soon as I was in ketosis and, 4 months later, I ran my personal best marathon time of 5½ h, which for me was astonishing. I started to notice when I adhered to my diet my breathing was less labored. Encouraged, I started to read more including a paper that showed ketones inhibit the NLRP3 inflammasome. I had also read about the NLRP3 and its role in COPD. I asked a respiratory professional whom I met at a conference, “what if we had a medication to inhibit NLRP3?” He said, “that could represent a remarkable step forward in the treatment and patient care.” That sealed it for me. I've been on a ketogenic diet ever since and, combined with my exercise, my respiration appears to be ever improving. It's unlikely I'll ever have completely normal breathing, but I'm so grateful to being trending upward, not downward. I hope my experience will encourage others to try a lifestyle that significantly improved my quality of life.

## Limitations

As with any retrospective *n* = 1 patient case, this report contains limitations that must be acknowledged. (i) First, the duration of time between the initiation of the KD and subsequent FEV_1_ and cytokine tests was ~2 years. It is plausible that other changes in the patient's lifestyle during this time contributed to his symptomatic and inflammatory improvements. While the patient attests that “I was surprised to find my breathlessness diminished as soon as I was in ketosis,” and that “when I adhered to my diet my breathing was less labored,” and that he ran his personal best marathon time 4 months after the initiation of his diet, these data are self-reported and/or subjective and should be taken as such. (ii) Second, and building on the above, there is the possibility of a placebo interaction. As the patient was encouraged about the possibility that a ketogenic diet could improve his athletic performance and symptoms, it is feasible that this optimism changed his exercise confidence, or other aspects of lifestyle, contributing to his improvements. It is also possible that his enthusiasm and optimism led him to order the direct-to-consumer cytokine panels at symptomatically favorable moments when inflammation might have been in a trough. Although he denies this was the case, and it seems unlikely that a placebo interaction could explain the full effect and its consistency (assuming the effect is genuine and due to the diet), it is an important caveat, nonetheless. (iii) Third, the patient represents a highly particular case of COPD, as most patients are not marathon runners and do not engage in intensive physical activities. It would therefore be premature to generalize the findings of this report to a wider COPD population. (iv) Finally, it would have been ideal to not only have more data timepoints but also more functional measures of lung function. Unfortunately, as this is a retrospective case, the data are limited to that which the patients and treating physician had available to us.

## Summary

We report on a case in which initiation and continuation of a ketogenic diet was associated with improvements in the lung function and inflammatory markers of a patient with COPD. As ketogenic diets have the potential to be anti-inflammatory diets, COPD is an inflammatory disorder, and ketogenic diets are being explored for an increasing array of inflammatory conditions, this case suggests ketogenic diets could have therapeutic potential in COPD and that more research is needed.

## Data Availability Statement

The original contributions presented in the study are included in the article/supplementary material, further inquiries can be directed to the corresponding author/s.

## Ethics Statement

Written, informed consent was obtained from the participant for the publication of this case report and any potentially-identifying information/images.

## Author Contributions

All authors listed have made a substantial, direct and intellectual contribution to the work, and approved it for publication.

## Conflict of Interest

RW receives funding from Philips Respiratory Care as Global Brand Ambassador. PB reports research funding from AstraZeneca and Boehringer Ingelheim and is an advisor to AstraZeneca, Boehringer-Ingelheim, Covis, Epi-Endo, Pieris, and Teva. BS has stock options in two companies that commercialize exogenous ketone products and is an inventor on patents that relate to ketone bodies. DD'A is an inventor on patents related to therapeutic applications of ketone bodies and co-owner of the company Ketone Technologies LLC, providing scientific consulting and public speaking on ketogenic therapies. The remaining author declares that the research was conducted in the absence of any commercial or financial relationships that could be construed as a potential conflict of interest.

## Publisher's Note

All claims expressed in this article are solely those of the authors and do not necessarily represent those of their affiliated organizations, or those of the publisher, the editors and the reviewers. Any product that may be evaluated in this article, or claim that may be made by its manufacturer, is not guaranteed or endorsed by the publisher.

## Abbreviations

COPD: Chronic obstructive pulmonary disease
FEV_1_: forced expiratory volume in 1 second
GM-CSF: granulocyte-macrophage colony stimulating factor
KD: ketogenic diet
IL-1: interleukin
NLRP3: NOD-LRR- and pyrin domain-containing protein 3
TNF-α: tumor necrosis factor-α
βHB: β-hydroxybutyrate.
